# High-Performance Nickel–Bismuth Oxide Electrocatalysts Applicable to Both the HER and OER in Alkaline Water Electrolysis

**DOI:** 10.1021/acsami.4c15514

**Published:** 2025-02-12

**Authors:** Seunghyun Jo, Byeol Kang, SiEon An, Hye Bin Jung, JunHwa Kwon, Hyunjun Oh, Jeonghyeon Lim, Pilsoo Choi, Jungho Oh, Ki-yeop Cho, Hyun-Seok Cho, MinJoong Kim, Joo-Hyoung Lee, KwangSup Eom, Thomas F. Fuller

**Affiliations:** † School of Materials Science and Engineering, Gwangju Institute of Science and Technology (GIST), 123 Cheomdangwagi-ro, Buk-gu, Gwangju 61005, Republic of Korea; ‡ Hydrogen Research Department, Korea Institute of Energy Research, 152 Gajeong-ro, Yuseong-gu, Daejeon 34129, Republic of Korea; § Department of Chemical and Biomolecular Engineering, Sogang Universiity, 35 Baekbeom-ro, Mapo-gu, Seoul 04107, Republic of Korea; ∥ School of Chemical and Biomolecular Engineering, Georgia Institute of Technology, Atlanta, Georgia 30332, United States

**Keywords:** water electrolysis, hydrogen evolution reaction, oxygen evolution reaction, electrocatalyst, nickel oxide, bismuth, hydrothermal

## Abstract

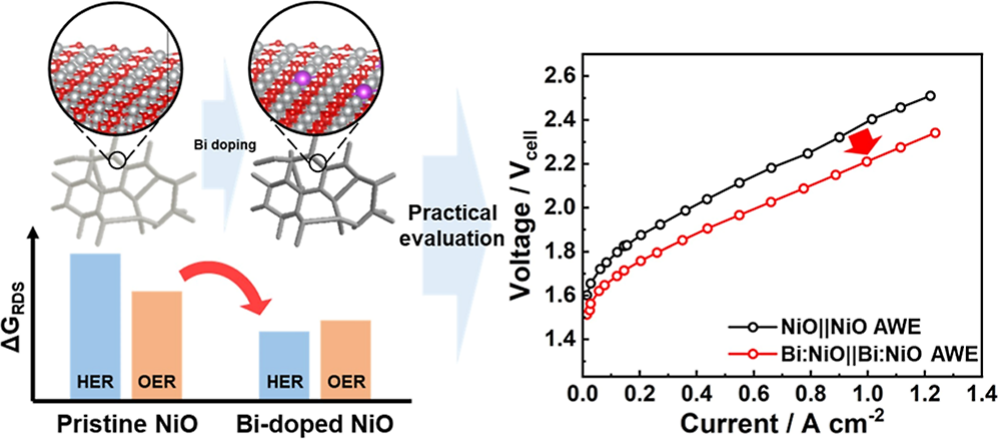

As an electrocatalyst for water electrolysis, nickel oxide (NiO) has received significant attention due to its cost-effectiveness and high reactivity among non-noble-metal-based catalytic materials. However, NiO still exhibits poor alkaline hydrogen evolution reaction (HER) and oxygen evolution reaction (OER) kinetics compared to conventional noble metal-based catalysts. This is because NiO has a strong interaction with protons for the HER and too low free energy of the OH* state, resulting in slower rate-determining step (RDS) kinetics for the OER. To address these issues, adding a dopant is suggested as an efficient method to modify the electron structure of the NiO electrocatalyst favorably for each reaction kinetics. In this context, we demonstrate that Bismuth (Bi), due to its higher electronegativity than that of Nickel (Ni), induces a positive charge on Ni sites. This enhances the catalytic activity by reducing the number of excessive cation interactions with the NiO electrocatalyst. Moreover, as the Bi ratio increases, the Ni reaction sites in NiO become more positively charged, and these changes in the electronic structure directly impact the free energy of the reaction mechanism. Particularly, it is confirmed that for the HER, Bi additives increase the proton-adsorbed free energy toward a near-zero value and, additionally, decrease the free energy difference of the second step considered as the RDS in the OER, as calculated by density functional theory. The positive effects of Bi in both the HER and the OER are demonstrated in practical electrochemical evaluations of half/single cells. Notably, the Bi-containing catalysts Bi05:NiO and Bi02:NiO exhibit remarkable alkaline HER and OER kinetics, showing performance improvements of 97.0% and 21.9%, respectively.

## 1 Introduction

Recently, as environmental pollution caused by fossil fuels has worsened, the necessity for eco-friendly fuels has been widely recognized.^1^ Among these, hydrogen fuel has received significant attention as a next-generation energy source owing to its high energy density and eco-friendliness, generating only water as a byproduct. However, current hydrogen production via steam reforming generates carbon dioxide, causing more environmental pollution than the use of fossil fuels.^2,3^ In contrast, water electrolysis, which induces the hydrogen evolution reaction (HER) and the oxygen evolution reaction (OER) from water using separate cathode and anode electrodes, is a perfectly eco-friendly method.^4,5^ However, the high activation energy of the HER and OER necessitates the use of electrocatalysts, and the high cost of these catalysts is the primary obstacle to achieving sufficient cost-competitiveness of hydrogen produced by water electrolysis compared to the other processes. Therefore, to overcome this barrier, it is essential to study the catalytic mechanisms of both the HER and OER and design suitable, low-cost catalytic materials.^6^

For instance, the mechanism of the HER in alkaline media is as follows
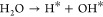
1
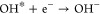
2
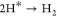
3

In this mechanism, the free energies of the initial (H_2_O, reactant of eq 1) and final (H_2_, product of eq 3) states are equal to 0 eV.^7,8^ Therefore, to minimize the free energy difference of the rate-determining step (RDS), the free energy difference of each intermediate should be close to 0 when using a catalyst. Pt/C composites are currently the most widely used catalytic materials for the HER because Pt has the most zero-like proton-adsorbed free energy among all homogeneous catalyst materials, resulting in the highest catalytic activity.^9,10^ However, its rarity on the earth’s surface, constituting only 3.7 × 10^–6^% of the crust, poses significant cost limitations.^11^ To address this issue, numerous studies on various nonprecious metal catalysts to substitute for Pt are underway. Among transition metals, nickel (Ni) has shown the highest theoretical and practical suitability for catalytic performance, potentially approaching that of Pt.^12,13^ However, focusing on the HER mechanism, nickel has a significantly negative proton-adsorbed free energy and exhibits a rapid adsorption step (eqs 1, 2) due to strong interactions with protons; thus, it makes the detachment of protons the RDS (eq 3).^14^ Therefore, the primary objective is to increase the proton-adsorbed free energy by altering the electronic structure of Ni.

On the other hand, the OER mechanism in an alkaline medium is as follows
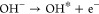
4
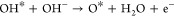
5
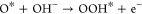
6
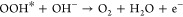
7

Since there is a difference of 4.93 eV in the free energy between the initial and final states of the OER, an ideal catalyst would equalize the free energy difference of each of the four reactions to 1.23 eV.^15^ Currently, the most commonly used catalysts are precious metal catalysts, such as IrO_2_, which are high-cost materials.^16,17^ Therefore, finding feasible alternatives to IrO_2_ among nonprecious metals is essential. Specifically, nickel oxide (NiO), one of the transition metal oxides, is a promising material in the electrocatalytic OER due to its theoretically highest catalytic activity among nonprecious catalysts.^18^ Nevertheless, NiO still has a hurdle to replace the IrO_2_, especially as focused on the mechanism of the alkaline OER, the step from the OH* state to the O* state becomes the RDS (eq 5), and NiO shows high activation energy of the RDS owing to low free energy of the OH* state.^19^ Therefore, it is necessary to engineer a change in the electronic structure to effectively reduce the free energy difference in the RDS for this reaction.

To modify the electronic structure of NiO and optimize it for the mechanisms of the HER and OER, one of the most effective strategies is doping with elements such as iron (Fe),^20−23^ zinc (Zn),^24−27^ copper (Cu),^28^ molybdenum (Mo),^29,30^ and cobalt (Co).^31−33^ Specifically, doping with atoms that have higher electronegativity than Ni helps reduce the activation energy of the RDS. When utilizing additives with higher electronegativity than nickel, Ni tends to donate electrons to the additive, resulting in a positive charge on the Ni reaction site and weakening the attraction between the Ni and cations.^34^ Consequently, positively charged Ni can reduce the free energy increment for proton desorption in the HER (eq 3) and for the adsorption of hydroxide ions from the OH* to O* phase in the OER (eq 5), which are the RDS for each respective reaction.^34,35^

However, the main drawback of NiO-based catalysts with added additives is their low durability. According to previous studies, the lifetime of NiO-based catalysts is approximately 10% that of noble metal-based catalysts, such as IrO_2_.^36^ The primary causes of this short lifespan are side reactions that lead to reaction site dissolution and phase segregation, both of which result in oxidative degradation. For NiO catalysts, activation to the β-NiOOH phase occurs prior to the OER, after which the reaction proceeds through the steps outlined in eqs 4–7. During this activation, oxidation is driven by OH^–^ ions, but when OH^–^ ions induce deactivation by proton abstraction rather than simple adsorption, NiO transforms into NiO_2_, leading to catalyst deactivation.^37^ It also affects NiO catalysts with additives. For instance, in NiFe-LDH catalysts, Fe serves as the primary reaction site; however, studies report that Fe undergoes dissolution via ionization after the step in eq 6, further compromising catalyst stability.^38,39^

In this study, bismuth (Bi) was utilized to develop a high-performance catalyst for the HER and OER. Bi, with a higher electronegativity of 2.02 than that of Ni (1.91), can positively charge Ni reaction sites, enhancing the catalytic activity of Ni by inducing high interactions with anions. Moreover, Bi not only has higher electronegativity than Ni but also exhibits stability without corrosion due to its reversible redox reactions between oxidation states 0, +2, and +3 under alkaline HER and OER conditions.^40,41^ Particularly in the case of the OER, the repeated adsorption of OH^–^ ions causes the oxidation state of the catalyst surface to fluctuate between Ni^2+^ and Ni^3+^ oxide states.^19^ In this context, the characteristic of the Bi additive directly impacts the catalyst’s durability as the reversible redox reactions help maintain the solid phase without phase transformation of the reaction site during the OER, unlike other additives that undergo deactivation due to side reactions. By preserving the solid phase, these reversible reactions can mitigate current loss resulting from side reactions, thereby enhancing durability. Despite these advantages, to the best of our knowledge, no work on Bi-containing NiO catalytic materials for water electrolysis has been reported thus far. Therefore, this study aims to fabricate Bi-containing NiO structures, analyze their alkaline HER and OER mechanisms related to the surface properties of the materials, and finally evaluate their practical feasibility in half-cells and a full single-cell system for water electrolysis.

## 2 Experimental Section

### 2.1 Synthesis of the Bi:NiO Electrocatalyst

Bismuth-doped nickel oxide (Bi:NiO) was synthesized using a hydrothermal process. First, 50 mM Ni(NO_3_)_2_, 0–10 mM Bi(NO_3_)_2_, 50 mM sodium dodecyl sulfate, and 1.5 g of urea were dissolved in a mixed solvent of 45 mL of ethylene glycol and 15 mL of deionized (DI) water. The solution was transferred into a Teflon-lined autoclave (80 mL), and nickel foam (NF) was dropped into the solution. The autoclave was heated to 120 °C for 10 h. After rinsing with DI water and ethanol, the Ni foam was annealed in a furnace in air. The furnace temperature was increased to 400 °C (heating rate of 5 °C min^–1^), and the sample was cooled to room temperature.

### 2.2 Catalyst Characterization

The morphology of the catalysts was examined by using field-emission scanning electron microscopy (FE-SEM, JEOL, JSM-7500F) and transmission electron microscopy (TEM, FEI, Tecnai G2 F30 S-Twin). The crystallinity of the Bi:NiO catalysts was investigated using X-ray diffraction (XRD, Rigaku, Smartlab) with Cu Kα radiation (λ = 1.5418 Å) at a scanning rate of 1.0° min^–1^ (2θ = 20° ∼ 80°). The electronic structure and surface atomic composition were analyzed by using X-ray photoelectron spectroscopy (XPS, Thermo Scientific, K-ALPHA+). The spectra were calibrated using the C 1s binding energy (284.5 eV).

### 2.3 Electrochemical Test

The electrochemical half-cell tests were conducted in 1 M KOH solutions using a potentiostat (Solartron, Solartron 1285A) with a standard 3-electrode system that used a platinum rod and a saturated calomel electrode (SCE) as the counter and reference electrodes, respectively. For the analysis of HER and OER activity, the polarization curves of the Bi:NiO catalysts were measured by linear sweep voltammetry with a window potential range of −0.9 ∼ −1.5 V vs SCE and 0–1 V vs SCE at a scan rate of 10 mV s^–1^. After the measurement, the potential of the polarization curve was calibrated to the reversible hydrogen electrode (RHE) and *iR*-corrected. The stability of the catalysts for the HER and the OER was investigated by chronopotentiometry at reductive and oxidative current densities of 10 mA cm^–2^. The electrochemical measurements were conducted by purging N_2_ gas at 25 °C and atmospheric pressure.

The alkaline water electrolysis single-cell test was conducted with the composition of NiO||NiO (employing NiO at both the cathode and anode) and Bi05:NiO||Bi02:NiO (employing Bi05:NiO and Bi02:NiO) at the cathode and anode, respectively. Then, the Zirfon diaphragm (thickness of 500 ± 50 μm and porosity of 55 ± 10%) was used as a separator membrane, and the electrolysis reaction was carried out in a 25 wt % KOH solution, which flows as a rate of 500 mL min^–1^, at 80 °C, as shown in Figure S1. Then, the galvanostatic electrochemical impedance spectroscopy (GEIS) was conducted at the current density of 50 and 200 mA cm^–2^ with a frequency range from 100 kHz to 10 mHz and an amplitude of 5.8 mA cm^–2^.

### 2.4 Computational Method

To determine the free energies associated with the Bi:NiO catalysts, we conducted first-principles density functional theory (DFT) calculations using the Vienna ab initio simulation package (VASP).^42−46^ We solved the Kohn–Sham equation employing a plane-wave basis with a 400 eV cutoff and projector-augmented wave pseudopotentials.^47,48^ To optimize the geometry and calculate the free energies, we applied a generalized gradient approximation by Perdew, Burke, and Ernzerhof to handle the exchange–correlation energy between electrons.^49^

The computational configurations of the Bi:NiO structures consist of five layers representing a fixed Bi:NiO bulk region and two surface layers with varying surface Bi ratios. All atomic positions in the two surface layers were optimized using a 6 × 7 × 1 *k*-point mesh until the energy difference between successive relaxation steps was less than 0.001 eV. To compute the change in the free energy (Δ*G*), we considered the contributions from the difference in the zero-point energy (Δ*E*(ZPE)) and entropy changes (Δ*S*).^50,51^ Furthermore, we considered the adsorption energies (Δ*E*) of hydrogen and hydroxide ions in the vicinity of the surface under both acidic and alkaline conditions, as indicated in Figure S2.

## 3 Results and Discussion

### 3.1 Characterizations

**Figure 1:**
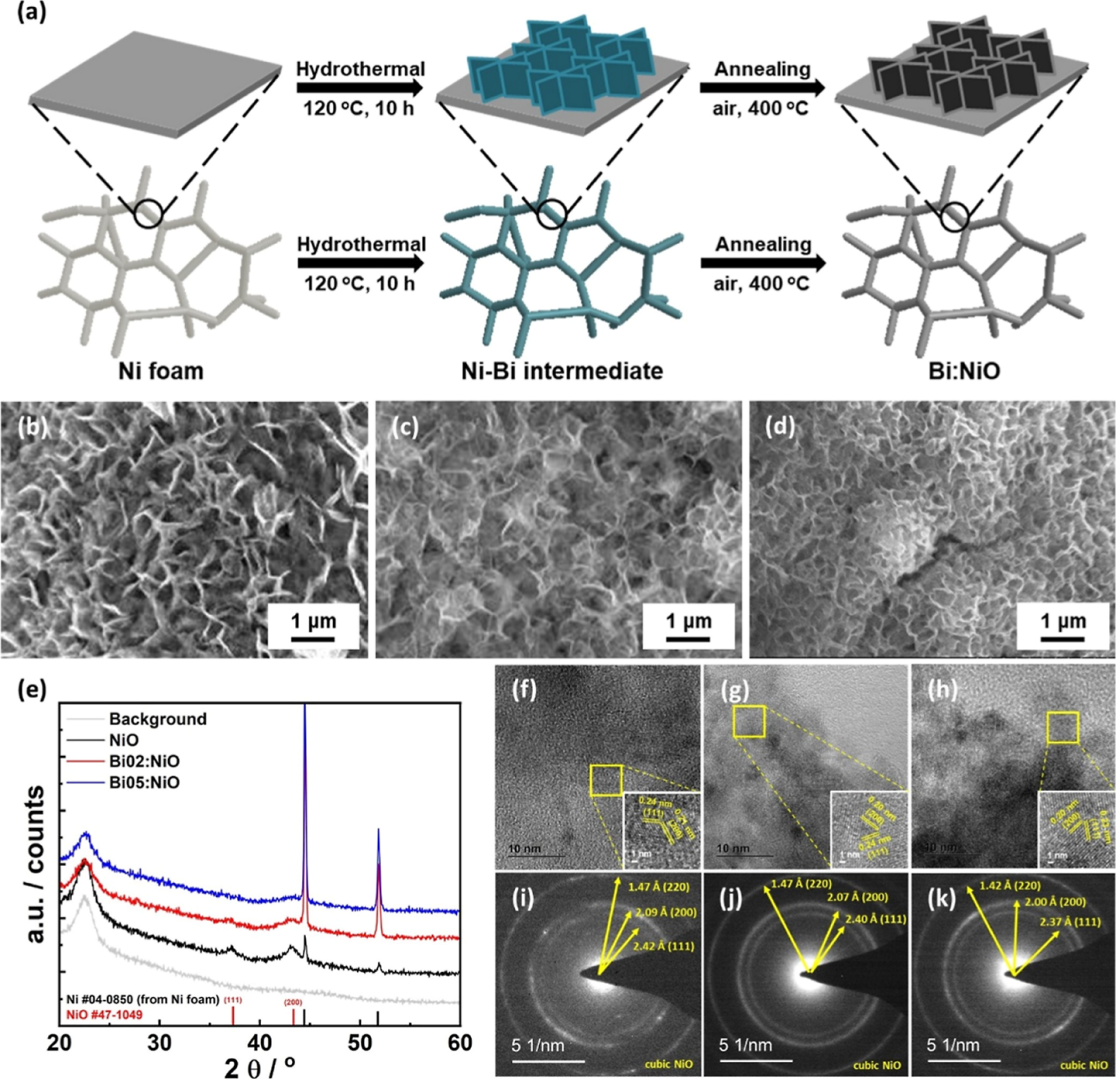
(a) Schematic illustration of the fabrication of Bi:NiO. Surface SEM images of (b) pristine NiO, (c) Bi02:NiO, and (d) Bi05:NiO. (e) XRD patterns of pristine NiO and Bi:NiO catalysts. TEM images and SAED patterns of (f,i) pristine NiO, (g,j) Bi02:NiO, and (h,k) Bi05:NiO.

The synthesis of the Bi:NiO catalyst is illustrated in Figure 1a, encompassing two sequential procedures: hydrothermal synthesis and annealing. The Bi:NiO catalyst was synthesized under hydrothermal conditions at 120 °C in the presence of ethylene glycol and Ni^2+^ ions. Subsequently, the intermediate of Bi:NiO, comprising Ni–Bi hydroxide and carbon compounds, was annealed at 400 °C in air to eliminate carbon-based byproducts and achieve the complete formation of the nickel oxide structure. The composition of the Bi additive was controlled by the concentration of the Bi precursor, with the resulting samples designated as “Bi00:NiO” (where “00” represents the millimolar concentration of the Bi precursor). The Bi concentrations in the Bi:NiO catalysts were set at 1, 2, 5, and 10 mM. This range was selected because at Bi precursor concentrations above 20 mM, phases other than Bi-doped NiO, specifically Bi_2_O_3_, began to form postsynthesis, resulting in the saturation of the doped Bi composition. Figures 1b–d and S3 present the surface SEM images of both pristine NiO and Bi:NiO catalysts with varying Bi compositions. Regardless of the Bi additive ratio, a layered structure was observed on the surfaces of both NiO and Bi:NiO catalysts.

The surface crystal structure of the catalysts was determined from the XRD patterns of powders separated via sonication. As shown in Figure 1e, the catalysts exhibited identical peaks at 37.2 and 43.3°, corresponding to NiO. Although peaks at 44.5 and 51.8° also revealed the crystal structure of Ni metal, they had limited relevance to the catalyst due to the presence of the Ni foam substrate. Then, the observed peaks of the Bi:NiO catalyst remained in the same positions, indicating that the crystal structure of the Bi:NiO catalysts is similar to that of NiO. Furthermore, TEM images and selected area electron diffraction (SAED) patterns revealed the microscopic crystal structure of Bi:NiO (Figures 1f–k and S4). In the TEM images and SAED patterns of pristine NiO (Figure 1f,i), lattice fringe spacings of 2.42, 2.09, and 1.47 Å were observed, corresponding to the (111), (200), and (220) facets of NiO, respectively. For the Bi:NiO catalysts, the TEM images and SAED patterns exhibited a similar tendency; however, they showed reduced lattice fringe spacings of 2.40, 2.07, and 1.47 Å for Bi02:NiO and 2.37, 2.00, and 1.42 Å for Bi05:NiO, as shown in Figures 1j,k, and S4, demonstrating that Bi was successfully doped into the NiO structure.

**Figure 2:**
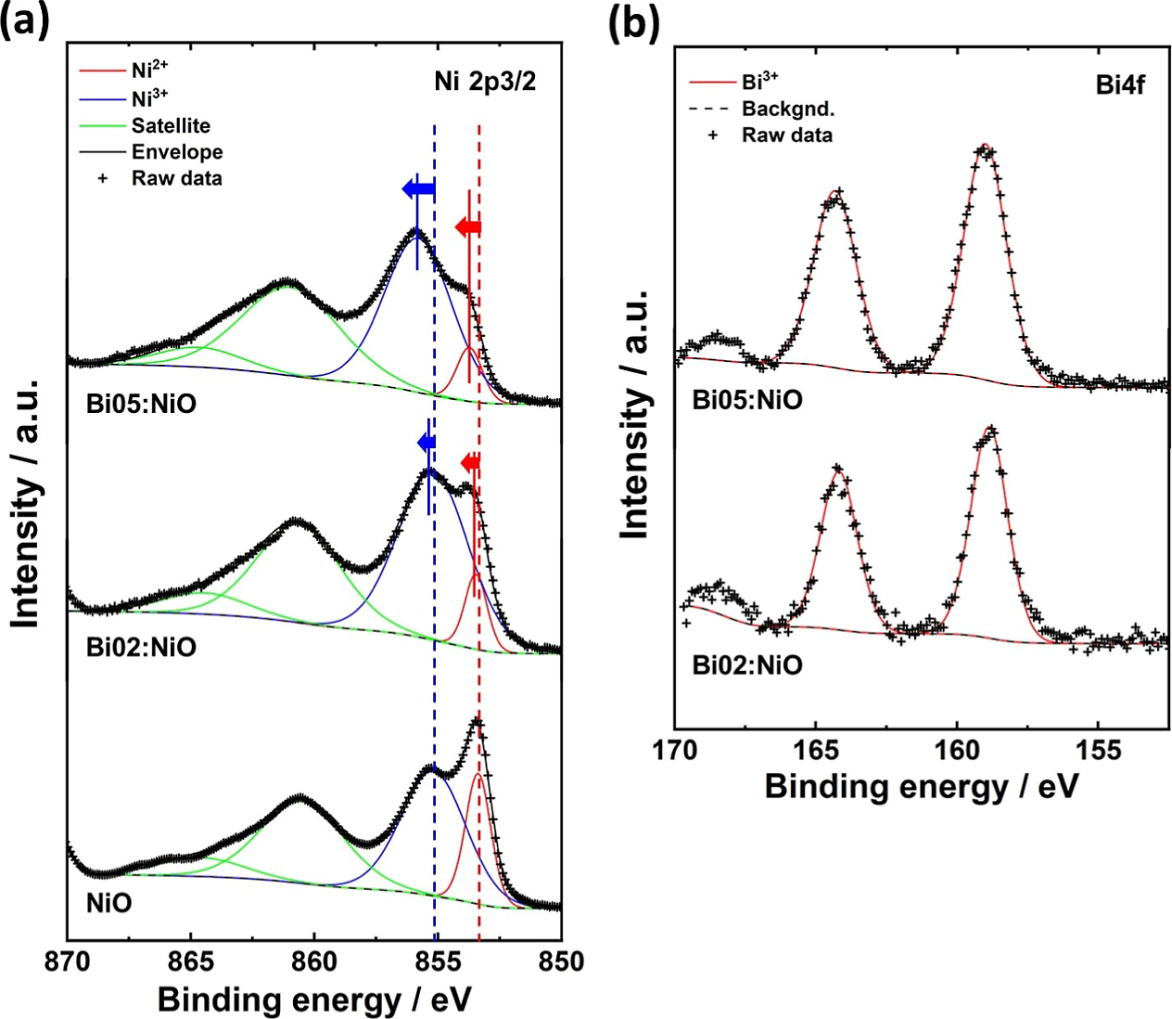
XPS spectra of the pristine NiO and Bi:NiO catalysts focused on the (a) Ni 2p3/2 orbital (binding energy from 850 to 867.5 eV) and (b) Bi 4f orbital (binding energy from 157 to 167 eV).

The electronic structure and surface composition of the catalysts were analyzed by using XPS. Figures 2 and S5 clearly demonstrate the differences in the spectra of the Ni 2p and Bi 4f orbitals affected by the amount of doped Bi. Specifically, Figure 2a shows the Ni 2p_3/2_ orbital, where the binding energies of Ni 2p_3/2_ appear at approximately 853 and 855 eV, corresponding to Ni^2+^ and Ni^3+^ oxides, respectively. As indicated in the XPS spectra of pristine NiO, peaks at 853.4 and 855.2 eV were attributed to the binding energies of Ni 2p_3/2_ from Ni^2+^ and Ni^3+^, respectively, suggesting the presence of NiO and NiOOH. Notably, these binding energy peaks shifted positively with an increase in the amount of Bi, indicating a modification in the electronic structure due to Bi doping. Compared to the Ni 2p_3/2_ peak of pristine NiO, Bi02:NiO exhibited a positive chemical shift of 0.09 and 0.17 eV for the binding energy corresponding to Ni^2+^ and Ni^3+^, respectively. In the case of Bi05:NiO, positive chemical shifts of 0.35 and 0.62 eV were observed for the binding energy of Ni^2+^ and Ni^3+^, respectively. Figure 2b displays the Bi 4f spectra, where the binding energies of Bi^3+^ were observed at approximately 158.7 and 164.0 eV. The binding energy of Bi^3+^ shows a slight shift with increasing Bi content. Moreover, the integrated spectra confirmed that the surface Bi ratio increased with the concentration of the Bi precursor; the Bi01:NiO, Bi02:NiO, Bi05:NiO, and Bi10:NiO catalysts contained 1.53, 2.96, 7.75, and 11.2 at % Bi, respectively.

### 3.2 DFT Calculations for the HER and OER

**Figure 3:**
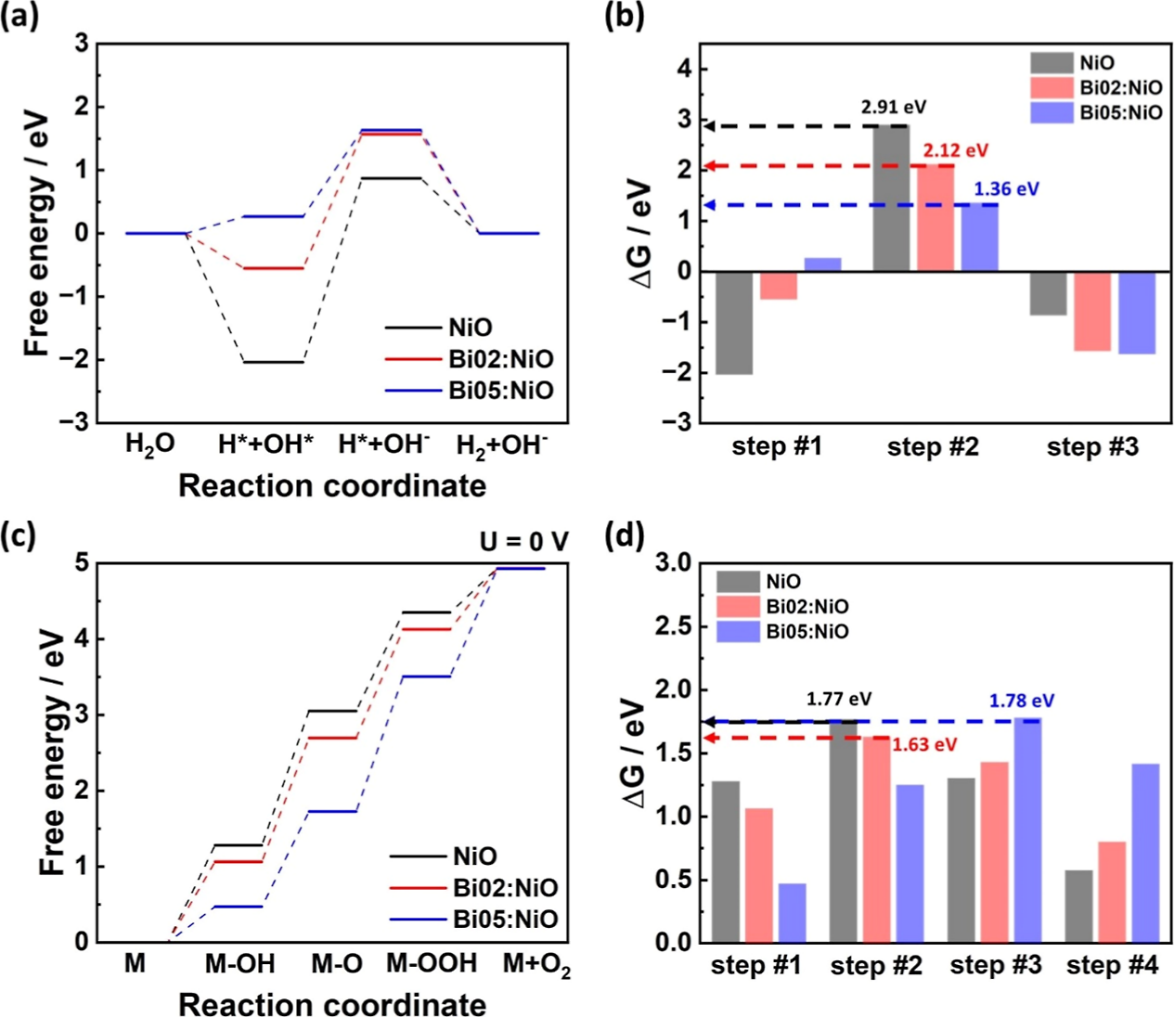
Free energy diagram investigated by DFT calculation of the catalysts for the (a,b) HER and (c,d) OER in alkaline media.

Based on the differences in the electron structure investigated through chemical measurements, a theoretical validation of the impact of the Bi dopant on catalytic performance in alkaline media was performed using DFT calculations. Given that the alkaline HER mechanism involves the dissociation of water molecules into adsorbed protons (H*) and hydroxide ions (OH*), its free energy profile is represented as a four-state diagram comprising H_2_O + e^–^, H* + OH* + e^–^, H* + OH^–^, and 1/2H_2_ + OH^–^ (Figure 3a). Moreover, the kinetics of the three steps between these states is investigated by analyzing the free energy difference profile (Figure 3b). Because low free energy difference means low activation energy of the step, a nearly zero difference in the free energy (Δ*G*) between each state is a crucial criterion for explaining the high catalytic activity. For the NiO catalyst, since the desorption of the hydroxide ion demonstrated the most positive free energy difference of 2.91 eV, it was inferred that hydroxide ion desorption constituted the RDS of the alkaline HER. On the other hand, after Bi doping, the free energies of both the H*+OH* state and the H*+OH^–^ state increased. In particular, with the increasing composition of Bi, the free energy of the H*+OH* state exhibited a more rapid rise than that of the H*+OH^–^ state. As a result, the Bi05:NiO catalyst displayed the smallest RDS free energy difference of 1.36 eV compared to those of other catalysts (Δ*G*_1/2H2+OH–_ – Δ*G*_H*+OH–_, Bi02:NiO: 2.12 eV).

On the other hand, the free energy profile of the OER in alkaline media involves the adsorption of four hydroxide ions (OH^–^), necessitating a five-state diagram: M, M–OH, M–O, M–OOH, and M + O_2_ (where M is the reaction site of the catalyst, Figure 3c). Because the free energy difference between the first state (M) and the final state (M + O_2_) is 4.92 eV, the ideal catalyst shows an equal free energy difference between each of the four steps, with the step having the highest free energy difference becoming the RDS of the alkaline OER. Therefore, the kinetics of four steps are investigated by the free energy difference profile, and the free energy difference (Δ*G*) of the RDS approximating 1.23 eV is a crucial criterion for explaining high catalytic activity (Figure 3d). For the pristine NiO catalyst, the second step (M–OH → M–O) demonstrated the most positive free energy difference of 1.77 eV between the reaction steps. In contrast, for the Bi:NiO catalyst, the free energy difference of the second step (M–OH → M–O), which conventionally served as the RDS, decreased with the composition of Bi, while the free energy difference of the third step (M–O → M–OOH) exhibited an increasing trend. Consequently, the Bi02:NiO catalyst maintained the second step (M–OH → M–O) as the RDS with a low free energy difference of 1.63 eV. However, for the Bi05:NiO catalyst, the third step (M–O → M–OOH) became the RDS with a higher free energy difference of 1.78 eV, resulting in decreased kinetics.

### 3.3 Catalytic Performances of the HER and OER

**Figure 4:**
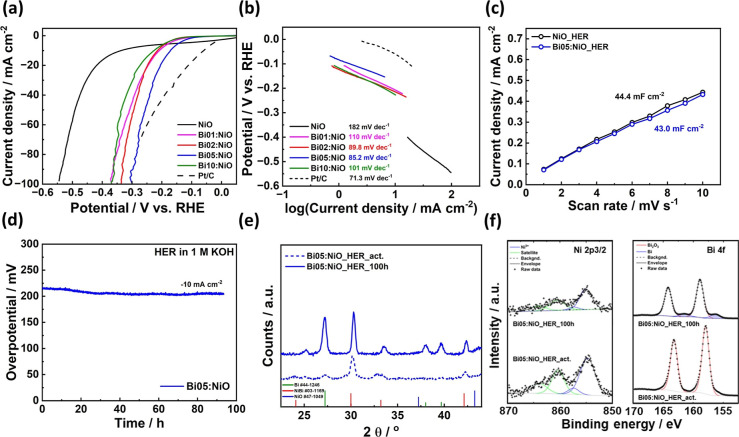
(a) *iR*-corrected polarization curves of pristine NiO and Bi:NiO catalysts for the HER in a 1 M KOH solution. (b) Corresponding Tafel plots of the HER polarization curve. The polarization curve and calculating Tafel slope of Pt/C were referred by Wu’s study.^54^ (c) Capacitance curves of the pristine NiO and Bi05:NiO. The curve was measured by CV from −0.3 to −0.2 V vs RHE in a 1 M KOH solution. (d) Chronopotentiometry test of the Bi05:NiO catalyst at a current density of −10 mA cm^–2^ for 100 h in a 1 M KOH solution. (e) XRD without background and (f) XPS spectra of the Bi05:NiO catalyst before and after the chronopotentiometry test. The electrochemical measurements were conducted at 25 °C.

Based on the promising theoretical catalytic activity of the Bi:NiO catalysts, the practical performance of catalysts was investigated by electrochemical measurements. The electrochemical evaluation of the Bi:NiO catalysts was assessed using a three-electrode system in a 1 M KOH solution at 25 °C. Figure 4 presents the electrochemical evaluation of the NiO and Bi:NiO catalysts for the HER. As shown in Figure 4a, the *iR*-corrected polarization curve indicates that Bi05:NiO exhibits the highest activity. To achieve a current density of 10 mA cm^–2^ for the HER, Bi05:NiO required the lowest cathodic overpotential of 171 mV among all prepared catalysts (pristine NiO: 337 mV; Bi01:NiO: 209 mV; Bi02:NiO: 215 mV; and Bi10:NiO: 227 mV). Additionally, concerning the Tafel slope, which elucidates intrinsic kinetics (Figure 4b), Bi05:NiO also demonstrated the smallest Tafel slope, measuring 85.2 mV dec^–1^, compared to all of the prepared catalysts (pristine NiO: 182 mV dec^–1^, Bi01:NiO: 110 mV dec^–1^, Bi02:NiO: 89.8 mV dec^–1^, and Bi10:NiO: 101 mV dec^–1^). Because the surface area can influence catalytic activity, the electrochemical surface area (ECSA) was measured using cyclic voltammetry in the non-Faradaic potential region. Although the Bi:NiO catalysts exhibited larger ECSAs depending on the amount of Bi (Figure S6), the ECSA of the Bi05:NiO catalyst was similar to that of the pristine NiO catalyst (Figure 4c). In this context, the addition of Bi alters the reaction site of NiO, resulting in a more appropriate electronic structure. To assess catalyst stability, a chronopotentiometry test was conducted at a reductive current density of 10 mA cm^–2^. As shown in Figure 4d, the overpotential remained stable for approximately 100 h, while the previous research studies pointed out the problem of the Ni-based electrocatalyst as low durability because of the fast dissolution under the HER condition.^12,52^ Bi05:NiO even showed an increase in efficiency during the durability test, suggesting an increase in the number of active reaction sites. According to the DFT calculation result, both Bi and Ni serve as adsorption sites for the HER activity in the Bi:NiO catalyst. In this point, the postactivation XRD spectra in Figure 4e reveal the formation of NiBi alloy crystalline structures alongside the initial NiO structure, with a notable increase in the NiBi alloy peak intensity following the chronopotentiometry test. Furthermore, the XPS spectra in Figure 4f indicate that while the binding energy of Ni^3+^ remains unchanged, there is an increase in Bi surface composition, evidenced by the emergence of a metallic Bi peak. Because the oxidation potential of Ni is lower than that of Bi under alkaline HER conditions, it suggests that the HER initiates Ni ionization and dissolution on the surface, which in turn enhances performance by increasing the number of active Bi sites.

**Figure 5:**
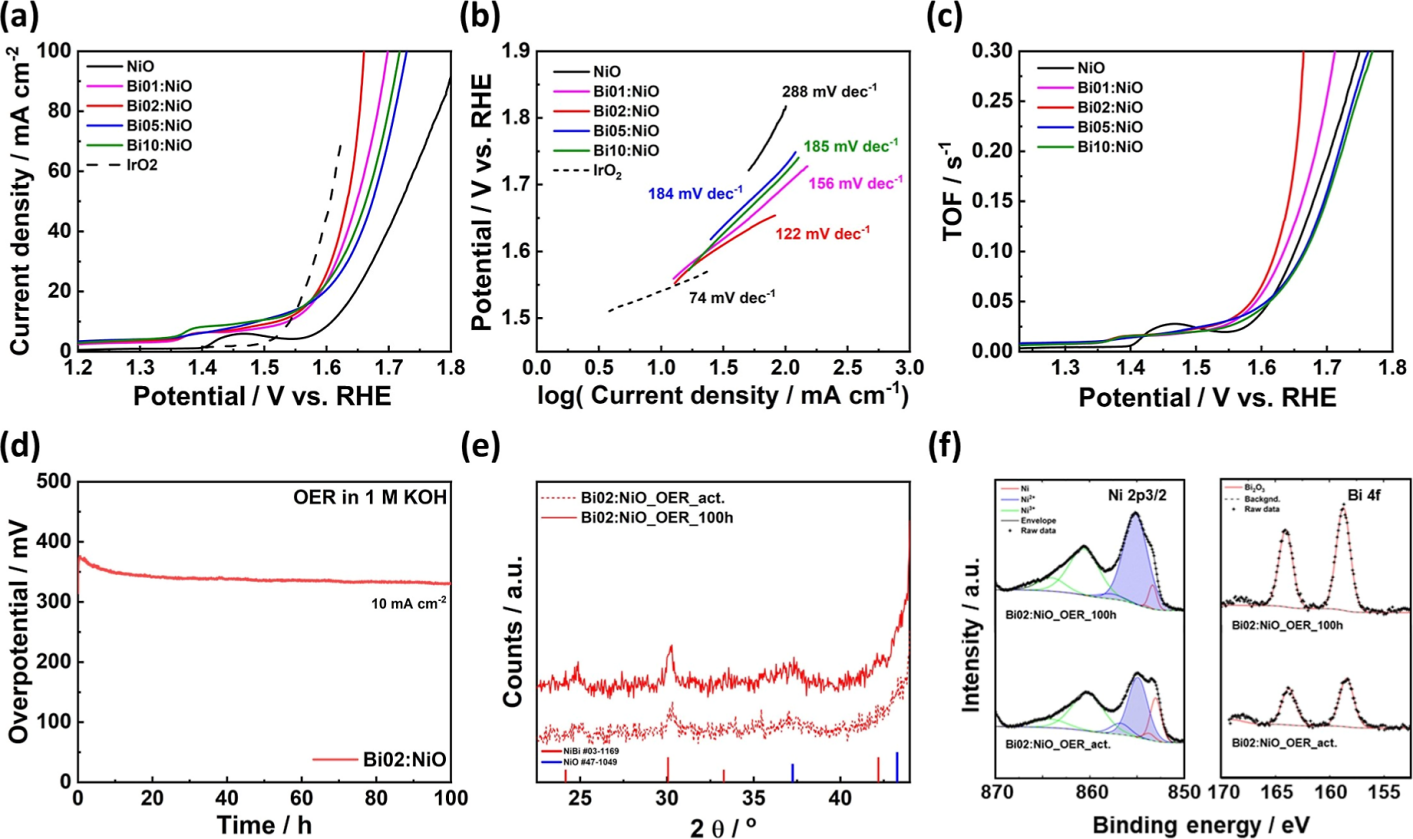
(a) *iR*-corrected polarization curves of pristine NiO and Bi:NiO catalysts for the OER in a 1 M KOH solution. (b) Corresponding Tafel plots of the OER polarization curve. The polarization curve and calculating Tafel slope of IrO_2_ were referred by Lin’s study.^55^ (c) Turnover frequency curves of pristine NiO and Bi:NiO catalysts calculated by the OER polarization curve. (d) Chronopotentiometry test of the Bi02:NiO catalyst at a current density of 10 mA cm^–2^ for 100 h in a 1 M KOH solution. (e) XRD without background and (f) XPS spectra of the Bi02:NiO catalyst before and after the chronopotentiometry test. The electrochemical measurements were conducted at 25 °C.

Figure 5 presents the electrochemical analysis of the catalysts to evaluate their performance in the OER in a 1 M KOH solution. According to the *iR*-corrected polarization curve (Figure 5a), NiO exhibited a higher catalytic activity after the addition of Bi. Consequently, the optimal composition of Bi differed from that of the catalyst used in the HER owing to the distinct mechanisms involved. In particular, Bi02:NiO required the lowest anodic overpotential of 402 mV to achieve a current density of 50 mA cm^–2^, while pristine NiO, Bi01:NiO, Bi02:NiO, and Bi10:NiO required 490, 418, 444, and 433 mV, respectively, to reach the same current density. To evaluate the intrinsic activity for the OER, the Tafel slope exhibited a trend similar to that of the polarization curve. As shown in Figure 5b, Bi02:NiO demonstrated a Tafel slope of 122 mV dec^–1^, which was the lowest value among all the prepared catalysts (pristine NiO: 288 mV dec^–1^, Bi01:NiO: 156 mV dec^–1^, Bi05:NiO: 184 mV dec^–1^, and Bi10:NiO: 185 mV dec^–1^). To evaluate the intrinsic HER activity of single catalytic sites quantitatively, the turnover frequency (TOF) was calculated using the following formula
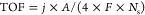
where *j* is the measured current density (A cm^–2^) at the *iR*-corrected overpotential (V), *A* is the area of the electrode (cm^2^), *F* is the Faraday constant (96485C mol^–1^), and *N*_s_ is the estimated number of reaction sites calculated via the CV plot for the redox reaction at various scan rates, as shown in detail in the Supporting Information and Figure S7. The calculated TOF results are shown in Figure 5c. As per the aforementioned calculations, the number of reaction sites is normalized for both the Ni and Bi metal centers, even though each metal center has different catalytic activities. Nevertheless, the TOF value for Bi02:NiO was 0.12 s^–1^ at an overpotential of 400 mV, while pristine NiO exhibited a TOF of 0.071 s^–1^. Furthermore, even if the limitation of Ni-based electrocatalysts from poor durability and short lifespan was reported by the previous studies,^36,53^Figure 5d displays the chronopotentiometry test results for pristine NiO and Bi02:NiO at a current density of 10 mA cm^–2^, with the overpotential of Bi02:NiO decreased during the durability test of 100 h. After the chronopotentiometry test, the crystallinity and oxidation states of Ni and Bi remained largely unchanged as compared with that of pristine Bi02:NiO, as shown in Figure 5e,f. Nevertheless, the XPS spectra show a relative increase in the Ni^3+^ and Bi peaks, indicating an increase in active Ni reaction sites and an elevated presence of Bi at Ni sites, both of which likely contribute to the improved catalytic performance.

### 3.4 Single-Cell Performance for Alkaline Water Electrolysis

Given the outstanding performance of the Bi:NiO catalysts for both the HER and the OER, Bi05:NiO was employed at the cathode and Bi02:NiO was employed at the anode in the alkaline electrolytic cell for overall water splitting. Because the nickel foam substrate served as a gas diffusion layer, there was no need for additional processes for the Bi:NiO electrodes. To prevent a short circuit between the cathode and anode, a Zirfon diaphragm (thickness of 500 ± 50 μm and porosity of 55 ± 10%) was used, and the electrolysis reaction was carried out in a 25 wt % KOH solution at 80 °C.

**Figure 6:**
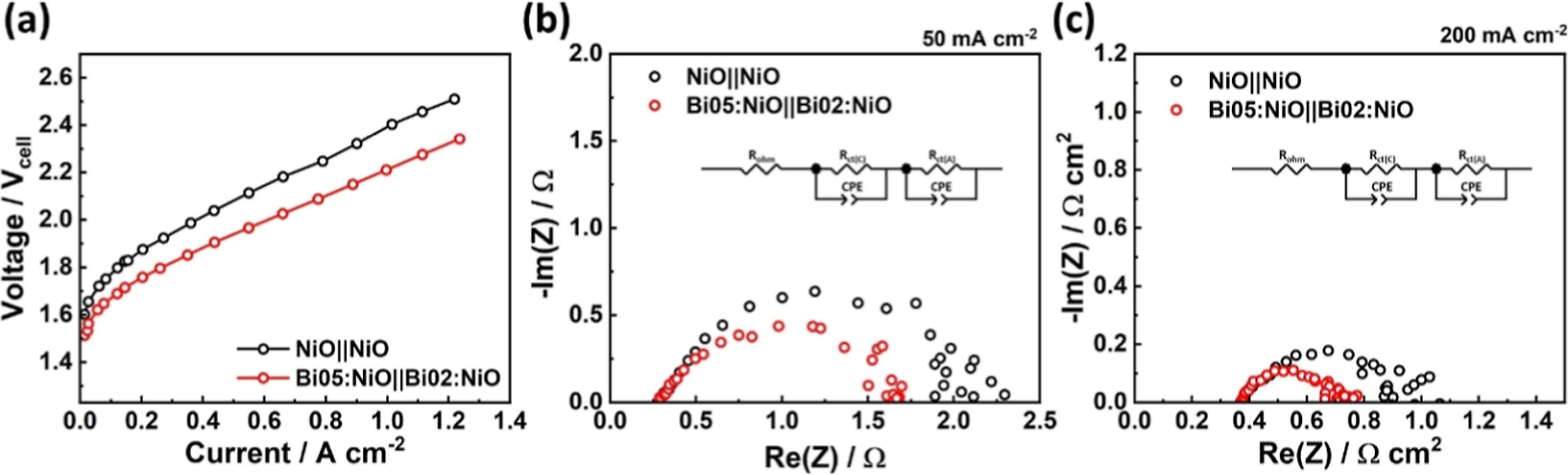
(a) *IV* curves of NiO||NiO and Bi05:NiO||Bi02:NiO alkaline water electrolysis single-cells. Nyquist plots of NiO||NiO and Bi05:NiO||Bi02:NiO alkaline water electrolysis single-cells measured by GEIS at (b) 50 mA cm^–2^ and (c) 200 mA cm^–2^.

As a result, Figure 6a displays the I–V curves for the NiO||NiO and Bi05:NiO||Bi02:NiO single-cells. Notably, the driving voltage required for the Bi05:NiO||Bi02:NiO cell to achieve 1A cm^–2^ was reduced by approximately 194 mV compared with that of the NiO||NiO cell. Electrochemical impedance spectroscopy was performed to analyze the factors contributing to current loss in the electrolysis cell (Figure 6b,c). After the addition of Bi, the charge transfer resistance (*R*_ct_) of the anode was 1.28 and 0.336 Ω cm^–2^ at the current density of 50 and 200 mA cm^–2^ (the NiO||NiO cell showed 1.60 and 0.550 Ω cm^2^), respectively, while the Ohmic resistance remained similar to that of the NiO||NiO cell.

## 4 Conclusions

In summary, we introduced Bi-doped NiO catalysts to enhance both the HER and the OER kinetics in alkaline media. By adjustment of the precursor concentration during hydrothermal synthesis, the Bi composition was optimized. While the crystal structure of NiO remained unchanged with Bi addition, a decrease in lattice spacing was observed with increasing Bi ratios. XPS analysis indicated that the electron structure of NiO became positively charged as the Bi ratio increased. Theoretical calculations suggested that Bi dopants reduce the activation energy of each RDS. For the HER, Bi doping increased the free energy of the H*+OH* state, resulting in a smaller free energy increment in the OH^–^ desorption step, with the Bi05:NiO catalyst exhibiting the lowest increment. For the OER, Bi doping decreased the free energy difference in the M–OH to M–O conversion step and increases it in the M–O to M–OOH step, with the Bi02:NiO catalyst achieving the most balanced free energy difference between these steps. As demonstrated by our calculations, the Bi05:NiO and Bi02:NiO catalysts exhibited the highest HER and OER activities and exceptional stability. For the HER, Bi05:NiO required the smallest overpotential of 171 mV to achieve a current density of 10 mA cm^–2^, with no efficiency decrease during 100 h of operation. For the OER, Bi02:NiO required the smallest overpotential of 402 mV to achieve a current density of 50 mA cm^–2^, with no efficiency decrease over 100 h of operation. Furthermore, in a single-cell test employing Bi05:NiO and Bi02:NiO as the cathode and anode, respectively, the cell voltage was reduced by 194 mV at a current density of 1 A cm^–2^ compared to that of the NiO||NiO cell. Accordingly, this work will provide new insights into the design of catalytic materials using suitable metallic additives in water electrolysis, based on theoretical calculations and practical evaluation using single cells.

## Supporting Information Available

Preparation of the NiO electrode; picture of (a) alkaline water electrolysis single cell and (b) water electrolysis station; geometric structures for DFT calculations of (a) pristine NiO, (b) Bi02:NiO, and (c) Bi05:NiO; surface SEM images of (b) Bi01:NiO and (d) Bi10:NiO; TEM images and SAED patterns of (a,c) Bi01:NiO and (b,d) Bi10:NiO; XPS spectra of the pristine NiO and Bi:NiO electrocatalysts focused on (a) the Ni 2p3/2 orbital (binding energy from 850 to 867.5 eV) and (b) Bi 4f orbital (binding energy from 157 to 167 eV); capacitance curves of the pristine NiO and Bi:NiO electrocatalysts;; CV plots of (a) pristine NiO, (b) Bi01:NiO, (c) Bi02:NiO, (d) Bi05:NiO, and (e) Bi10:NiO with a potential window of −0.4 to 0.6 V vs SCE and a scan rate of 5 to 50 mV s^–1^ in a 1 M KOH solution; current density–scan rate plots and calculated capacitance at the (f) Faradaic region; and detailed processes for calculating the number of reaction sites (*N*_s_) and TOF (PDF)
