# Supplementary material for: High-Performance Nickel–Bismuth Oxide Electrocatalysts Applicable to Both the HER and OER in Alkaline Water Electrolysis
Source: ACS Appl Mater Interfaces. 2025 Feb 12;17(8):11946–55. doi: 10.1021/acsami.4c15514 (PMC11873901; doi:10.1021/acsami.4c15514)
Supplement: Supplementary file 1 — am4c15514_si_001.pdf [file am4c15514_si_001.pdf]

Supporting Information

High-performance nickel-bismuth oxide electrocatalysts applicable to both HER and OER in alkaline water electrolysis

Seunghyun Jo[1], Byeol Kang[1], Si Eon An[2], Hye Bin Jung[3], JunHwa Kwon[1], Hyunjun Oh[1], Jeonghyeon Lim[1], Pilsoo Choi[1], Jungho Oh[1], Ki-yeop Cho[1], Hyun-Seok Cho[3], MinJoong Kim[2], Joo-Hyoung Lee[1]*, KwangSup Eom[1,4]*, and Thomas F. Fuller[4]*

[1]School of Materials Science and Engineering, Gwangju Institute of Science and Technology (GIST), 123 Cheomdangwagi-ro, Buk-gu, Gwangju, Republic of Korea

[2]Hydrogen Research Department, Korea Institute of Energy Research, 152 Gajeong-ro, Yuseong-gu, Daejeon 34129, Republic of Korea

[3]Department of Chemical and Biomolecular Engineering, Sogang Universiity, 35 Baekbeom-ro, Mapo-gu, Seoul 04107, Republic of Korea

[4]School of Chemical and Biomolecular Engineering, Georgia Institute of Technology, Atlanta, GA 30332, USA

* Corresponding author: E-mail: mj.kim@kier.re.kr (MinJoong Kim), jhyoung@gist.ac.kr (Joo-Hyoung Lee), keom@gist.ac.kr (KwangSup Eom)

ADDITIONAL INFORMATION

Preparation of the NiO electrode

Nickel oxide (NiO) was synthesized using a hydrothermal process. First, 50 mM $Ni(NO_3)_2$, 50 mM sodium dodecyl sulfate, and 1.5 g Urea were dissolved in a mixed solvent of 45 mL ethylene glycol and 15 mL deionized (DI) water. The solution was transferred into a Teflon-lined autoclave (80 mL), and nickel foam (NF) was dropped into the solution. The autoclave was heated to 120 ^{o}C for 10 h. After rinsing with DI water and ethanol, the Ni foam was annealed in a furnace in air. The furnace temperature was increased to 400 ^{o}C (heating rate of 5 ^{o}C min^{-1}) and cooled to room temperature.

Supplementary Figures and Tables

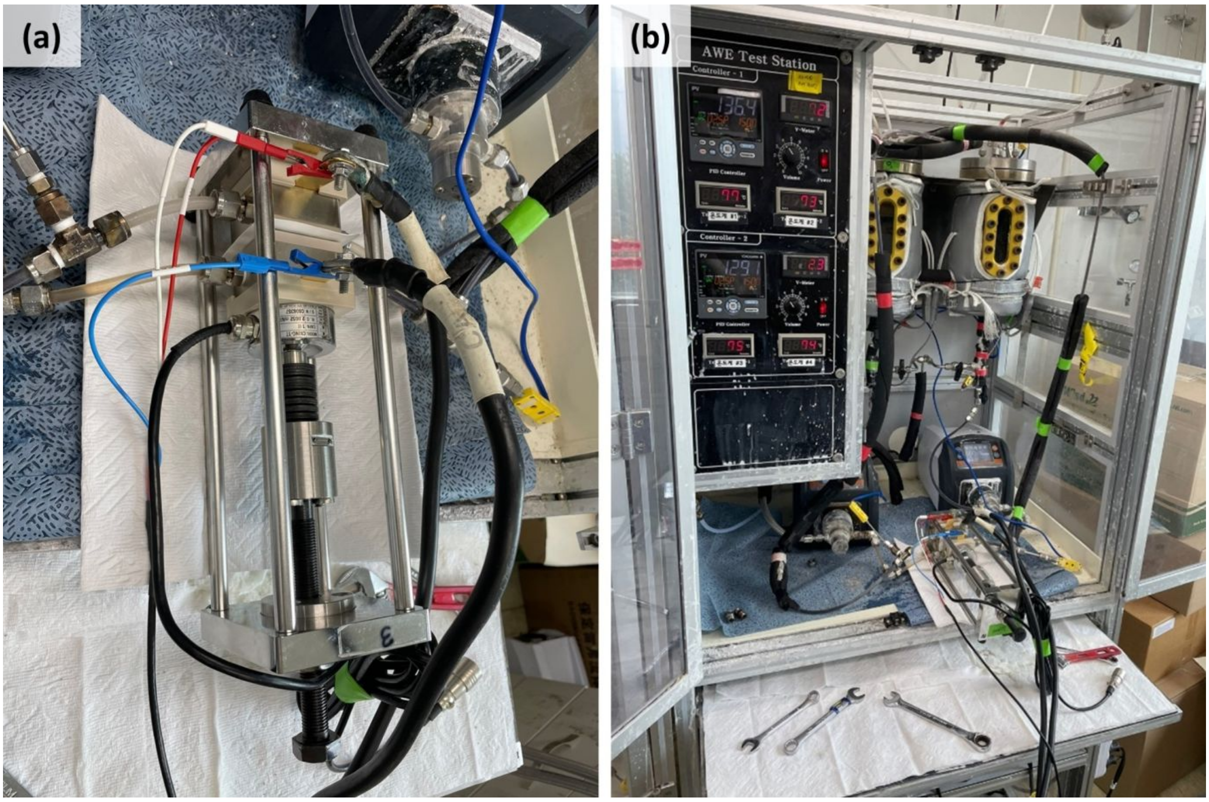

Figure S1. Picture of (a) alkaline water electrolysis single cell and (b) water electrolysis station.

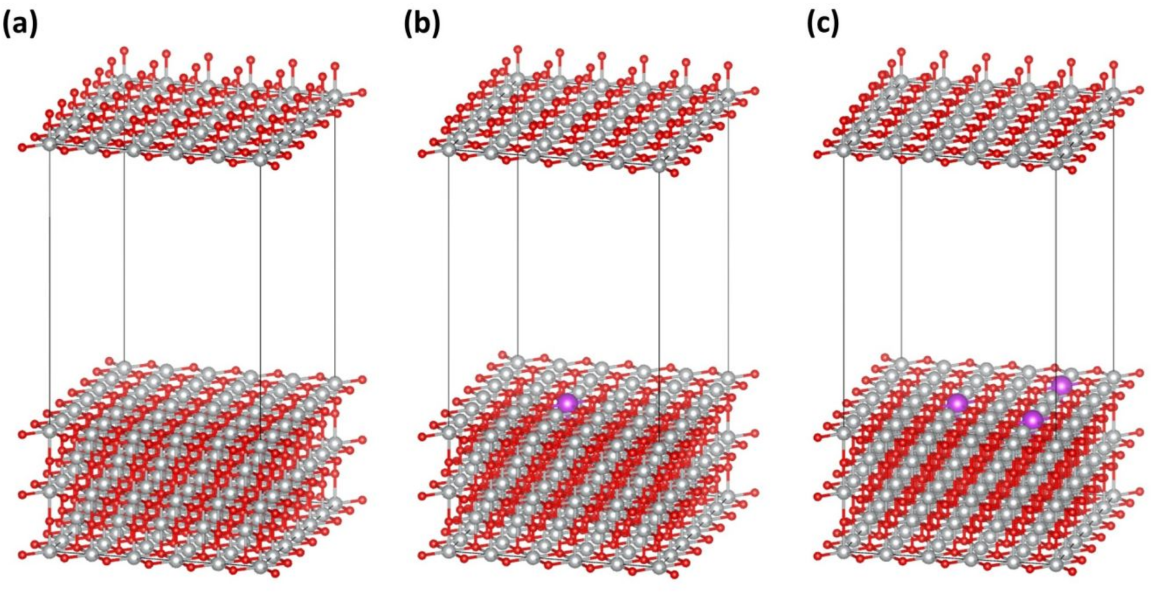

Figure S2. Geometric structures for DFT calculations of (a) Pristine NiO, (b) Bi02:NiO, and (c) Bi05:NiO.

For the generation of the Bi:NiO catalysis structures, we used the Alloy-Theoretic Automated Toolkit (ATAT) package to find the most stable special quasi-random structure (SQS).[1-5] After geometrical optimization of the Bi:NiO structures, hydrogen atoms and hydroxide ions are added and structurally relaxed, including two surficial layers. We subsequently calculated the adsorption energies of hydrogen atoms from

$$\Delta E(H^*) = \frac{1}{n}\left(E(M + nH^*) - E(M) - \frac{n}{2}E(H_2)\right)$$

$$\Delta E_{TS}(H^* + OH^*) = E(M + H^* + OH^*) - E(M + \frac{1}{2}H_2 + OH^-)$$

$$\Delta E_{TS}(OH^*) = E(M + H^* + OH^*) - E(M + H^* + OH^-)$$

$$\Delta E_{TS}(H^*) = \Delta E_{TS}(H^* + OH^*) - \Delta E_{TS}(OH^*)$$

in the alkaline reaction.[6]

Then, the ΔG for the adsorbed proton was calculated by the following equation:

$$\Delta G(H^*) = \Delta E(H^*) + \Delta E(ZPE) - T\Delta S$$

where ΔE(ZPE) is the difference in zero-point energy between the adsorbate and the gas phase and ΔS is the entropy of adsorption of 1/2 H_2 (ΔS = -1/2 $S(H_2)$).[6] According to the formal research, ΔE(ZPE) and TΔS are calculated to be 0.04 and -0.20 eV in 0.035 bar and 300K, respectively, thus ΔG(H*) and ΔE(H*) show:

$$\Delta G(H^*) = \Delta E(H^*) + 0.24\ eV$$

On the other hand, the ΔG for the transition site needs to find the coordination between adsorbed proton and hydroxide. The relation between ΔG(OH*) and ΔE(OH*) shows following relation when apply similar process with proton.[6]

$$\Delta G(OH^*) = \Delta E(OH^*) + 0.35\ eV$$

To account for the effect of solvation and alkali metal cation in electrolyte, it is necessary to reproduce the activation energy which is shifted by linear fit, then the adsorption energy of transition state was calculated as following.[7]

$$\Delta G(TS) = 0.72\Delta G_{TS}(H^*) + 0.51\Delta G_{TS}(OH^*) + 0.38$$

When the equation between ΔG and ΔE substituted, the equation between ΔG(TS) and adsorption energy of proton and hydroxide is derived as following equation.

$$\Delta G(TS) = 0.72\Delta E_{TS}(H^*) + 0.51\Delta E_{TS}(OH^*) + 0.73$$

As a calculation of oxygen evolution reaction, we calculated the adsorption energy of hydroxide ion from following eq. (10) and the ΔG for the adsorbed hydroxide ion was calculated by the following.

$$\Delta E(OH^*) = E(M + OH^*) - E(M + OH^-)$$

Finally, the ΔG for each step of OER was calculated by the following relation.[8]

$$\Delta G(O^*) = 0.64\Delta G(OH^*) + 0.95\ eV$$

$$\Delta G(OOH^*) = -0.59\Delta G(OH^*) + 2.06\ eV$$

$$\Delta G(O^2) = -1.04\Delta G(OH^*) + 1.91\ eV$$

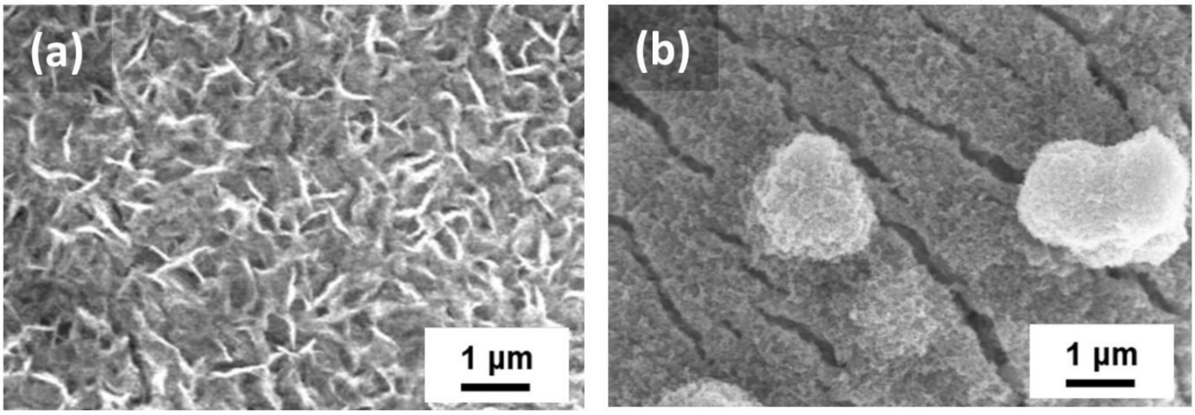

Figure S3. Surface SEM images of (a) Bi01:NiO and (b) Bi10:NiO.

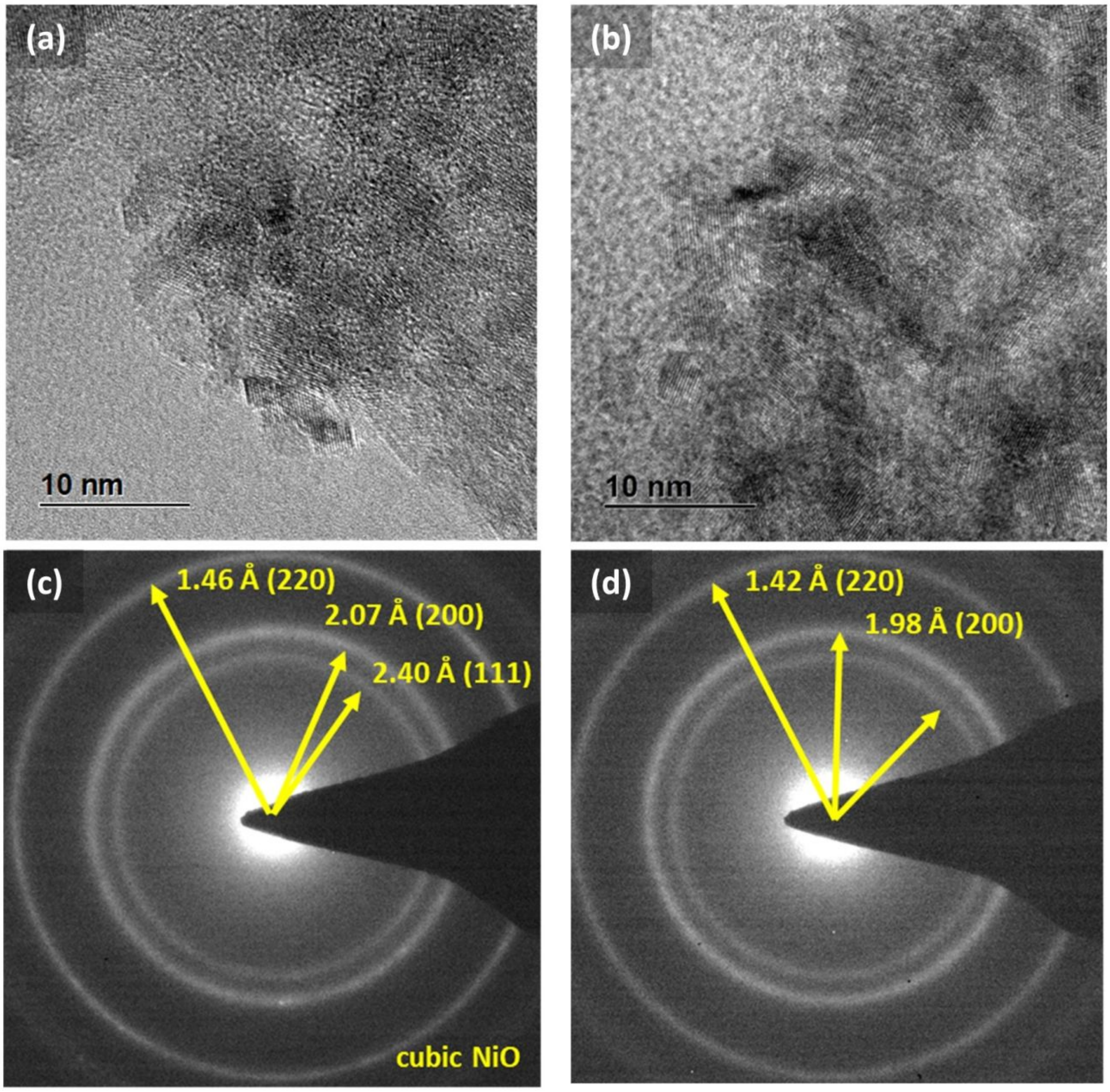

Figure S4. TEM images and SAED patterns of (a, c) Bi01:NiO and (b, d) Bi10:NiO.

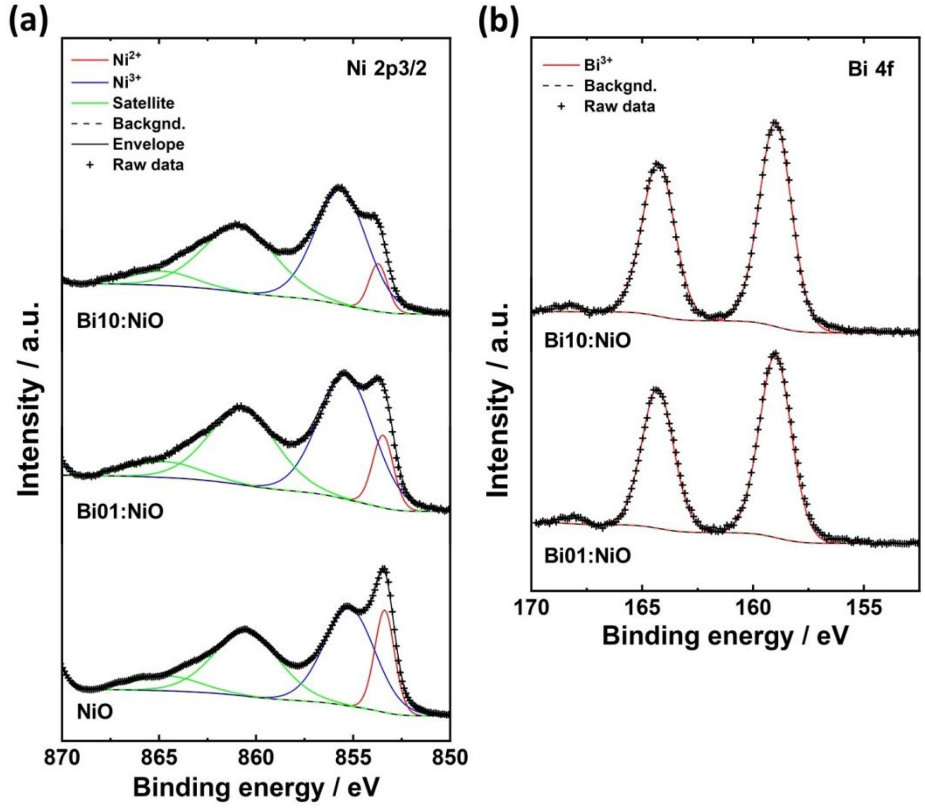

Figure S5. XPS spectra of the pristine NiO and Bi:NiO electrocatalysts focused on (a) the Ni $2p_{3/2}$ orbital (binding energy from 850 to 867.5 eV) and (b) Bi 4f orbital (binding energy from 157 to 167 eV).

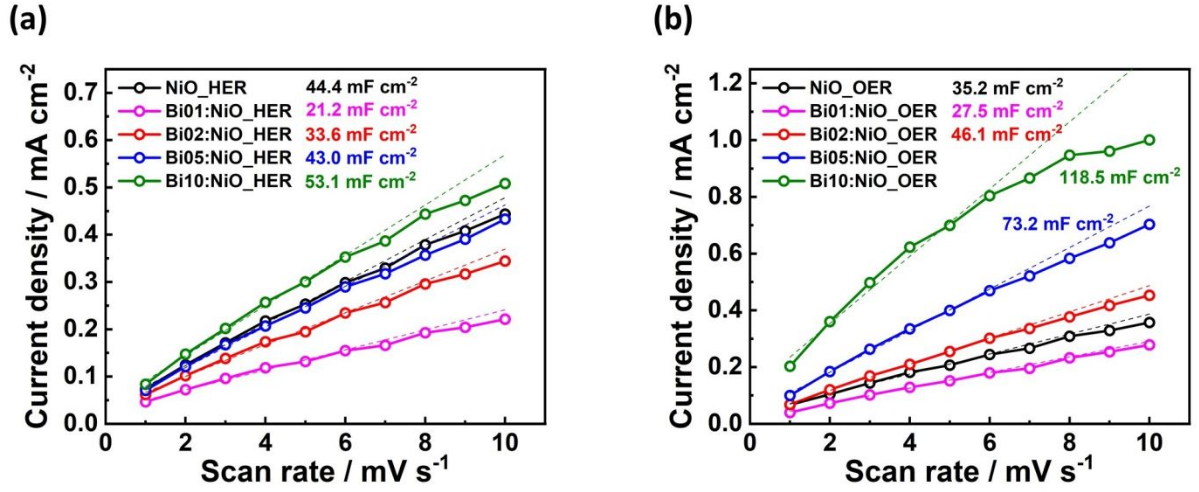

Figure S6. Capacitance curves of the pristine NiO and Bi:NiO electrocatalysts. The curve was measured by CV from -0.3 to -0.2 V vs. RHE in 1 M KOH solution. The measurements were conducted after the activation for (a) HER and (b) OER.

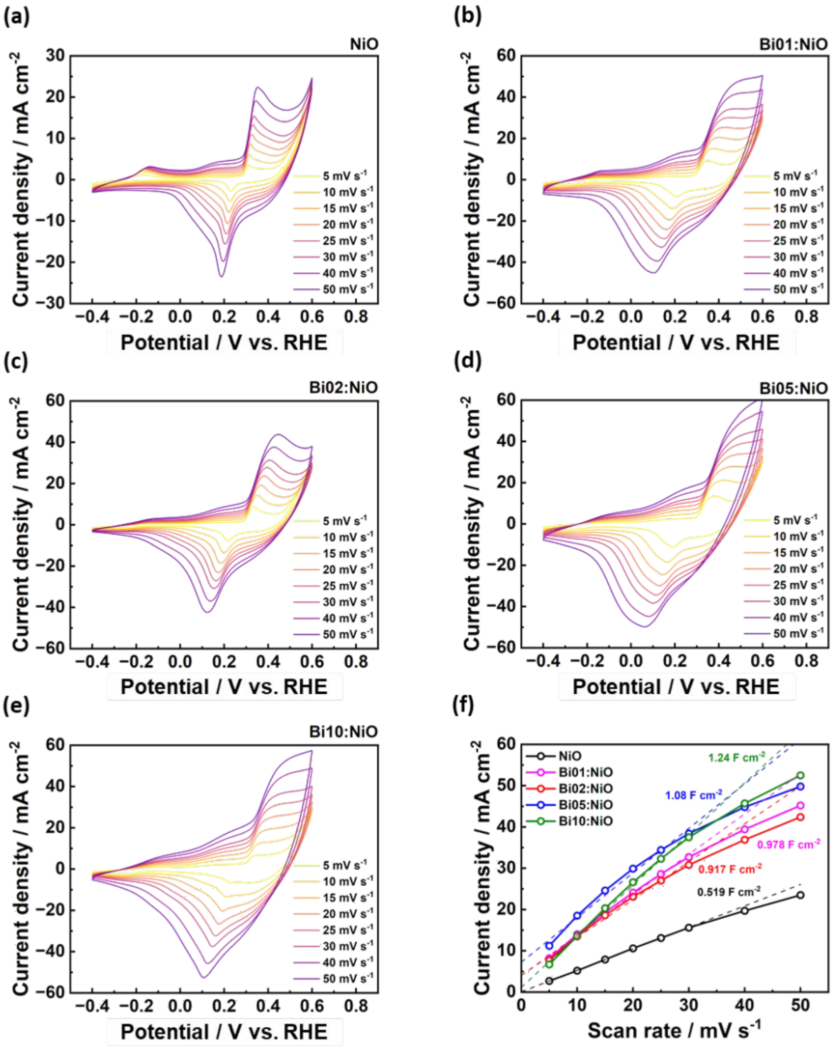

Figure S7. CV plots of (a) Pristine NiO, (b) Bi01:NiO, (c) Bi02:NiO, (d) Bi05:NiO, and (e) Bi10:NiO with a potential window of -0.4 to 0.6 V vs. SCE and a scan rate of 5 to 50 mV s^{-1} in 1 M KOH solution. Current density – scan rate plots and calculated capacitance at (f) faradaic region.

Number of reaction site (N_s)

The number of reaction sites can be calculated using the following method. First, to quantify the amount of OH^- involved in the redox reaction, the Ni^{2+}/Ni^{3+} redox peak current density is

measured at various scan rates (**Figure S7a-e**), and the results are plotted as shown in **Figure S7f**. Under the experimental conditions, the bulk concentration is sufficiently low to be considered negligible, allowing the current (i) to be derived from the change in the concentration of the oxidized species, as described by

$$i = -nFV\left[\frac{dC_{ox}(t)}{dt}\right]$$

where n, F, V, and t are, respectively, number of transfer electron, Faraday constant (96485 C mol^{-1}), volume, and time. Then, following equation was derived from the Nernst equation.

$$E = E^0 + \frac{RT}{nF} ln\frac{C_{ox}(t)}{C_{red}(t)}$$

In the above equation, E^0 and C(t) represent the potential of the redox species and the concentration of the redox product over time, respectively. Since the total amount of substance involved in the reaction remains constant, the sum of $C_{ox}(t)$ and $C_{red}(t)$ is also constant. As defining this total concentration as C_T, it can be substituted into formal as shown in following equation.

$$C_T = C_{ox}(t) + C_{re}(t)$$

$$C_{ox}(t) = \frac{C_T exp\left[\frac{nF}{RT}(E-E^0)\right]}{1+exp\left[\frac{nF}{RT}(E-E^0)\right]}$$

Then, following equation can be driven,

$$i = \frac{n^2F^2VC_Tv}{RT}\frac{exp\left[\frac{nF}{RT}(E-E^0)\right]}{\left\{1+exp\left[\frac{nF}{RT}(E-E^0)\right]\right\}^2}$$

where v represents the scan rate. Since E is equal to E^o at peak current and VC_T can be replaced by AN_s, following equation can be driven.

$$i = \frac{n^2F^2AN_s v}{4RT}$$

Finally, by rearranging the equation for N_s, a relationship between N_s and the slope of the current density (j) versus scan rate plot is obtained.

$$N_s = \frac{4\mathrm{RT}}{n^2F^2} \cdot \frac{j}{v}$$

In this point, the N_s values for the pristine NiO, Bi01:NiO, Bi02:NiO, Bi05:NiO, and Bi10:NiO catalysts were calculated from **Figure S7f**, resulting in 3.5 x 10^{-8}, 6.5 x 10^{-8}, 6.1 x 10^{-8}, 7.2 x 10^{-8}, and 8.3 x 10^{-8} mol cm^{-2}, respectively.

Turn-over frequency (TOF)

TOF represents the number of O_2 molecules generated per active site per unit time. Thus, the current density at a specific voltage obtained from the polarization curve and the previously calculated N_s exhibit the relationship expressed in following equation.

$$TOF = j \cdot \frac{A}{4FN_s}$$

Finally, it can be derived:

$$TOF = j \cdot \frac{An^2F}{16\mathrm{RT}} \cdot \left(\frac{j}{v}\right)^{-1} = j \cdot \frac{AF}{\mathrm{RT}} \cdot \left(\frac{j}{v}\right)^{-1} \text{ for OER.}$$

References

(1) van de Walle, A.; Tiwary, P.; de Jong, M.; Olmsted, D. L.; Asta, M.; Dick, A.; Shin, D.; Wang, Y.; Chen, L. Q.; Liu, Z. K. Efficient stochastic generation of special quasirandom structures. *Calphad* **2013,** *42*, 13-18, DOI: https://doi.org/10.1016/j.calphad.2013.06.006.

(2) van de Walle, A. Multicomponent multisublattice alloys, nonconfigurational entropy and other additions to the Alloy Theoretic Automated Toolkit. *Calphad* **2009,** *33* (2), 266-278, DOI: https://doi.org/10.1016/j.calphad.2008.12.005.

(3) Walle, A. v. d.; Asta, M. Self-driven lattice-model Monte Carlo simulations of alloy thermodynamic properties and phase diagrams. *Modelling and Simulation in Materials Science and Engineering* **2002,** *10* (5), 521-538, DOI: 10.1088/0965-0393/10/5/304.

(4) van de Walle, A.; Ceder, G. Automating first-principles phase diagram calculations. *Journal of Phase Equilibria* **2002,** *23* (4), 348, DOI: 10.1361/105497102770331596.

(5) van de Walle, A.; Asta, M.; Ceder, G. The alloy theoretic automated toolkit: A user guide. *Calphad* **2002,** *26* (4), 539-553, DOI: https://doi.org/10.1016/S0364-5916(02)80006-2.

(6) Nørskov, J. K.; Bligaard, T.; Logadottir, A.; Kitchin, J. R.; Chen, J. G.; Pandelov, S.; Stimming, U. Trends in the Exchange Current for Hydrogen Evolution. *Journal of The Electrochemical Society* **2005,** *152* (3), J23, DOI: 10.1149/1.1856988.

(7) McCrum, Ian T.; Koper, Marc T. M. The role of adsorbed hydroxide in hydrogen evolution reaction kinetics on modified platinum. *Nature Energy* **2020,** 5 (11), 891-899, DOI: 10.1038/s41560-020-00710-8.

(8) Dionigi, F.; Zeng, Z.; Sinev, I.; Merzdorf, T.; Deshpande, S.; Lopez, Miguel B.; Kunze, S.; Zegkinoglou, I.; Sarodnik, H.; Fan, D.; Bergmann, A.; Drnec, J.; Araujo, J. F.; Gliech, M.; Teschner, D.; Zhu, J.; Li, W.-X.; Greeley, J.; Cuenya, B. R.; Strasser, P. In-situ structure and

catalytic mechanism of NiFe and CoFe layered double hydroxides during oxygen evolution. *Nature Communications* **2020,** 11 (1), 2522, DOI: 10.1038/s41467-020-16237-1.
